# Characterization of a Robust 3D- and Inkjet-Printed Capacitive Position Sensor for a Spectrometer Application †

**DOI:** 10.3390/s19030443

**Published:** 2019-01-22

**Authors:** Lisa-Marie Faller, Martin Lenzhofer, Christina Hirschl, Martin Kraft, Hubert Zangl

**Affiliations:** 1 Institute for Smart System Technologies, Sensors and Actuators Department, Alpen-Adria-Universität Klagenfurt, 9020 Klagenfurt, Austria; hubert.zangl@aau.at; 2 Carinthia Tech Research AG, High Tech Campus, 9524 Villach, Austria; martin.lenzhofer@ctr.at (M.L.); christina.hirschl@ctr.at (C.H.); martin.kraft@ctr.at (M.K.)

**Keywords:** capacitive sensor, nanometer position measurement, inkjet-printing, 3D-printed metals, additive manufacturing

## Abstract

An inkjet- and 3D-printed capacitive sensor system with an all-digital and flexible sensor read-out hardware is reported. It enables spectrometer devices with significantly reduced device outlines and costs. The sensor is developed as multilayer inkjet-printed electrode structure on a 3D-printed copper housing. Very high required position resolutions of $res_{pos}<50\;\mathrm{nm}$ and a wide measurement range of $r_{m}$ = 1000 μm at an offset of $d_{0}$ = 1000 μm in the considered spectrometers motivate this work. The read-out hardware provides high sampling rates of up to $r_{s}\approx 10\;\mathrm{ns}$ and enables the generation of trigger signals, i.e., the mirror control signal, without a time lag. The read-out circuitry is designed as a carrier frequency system, which enables flexible choices of bandwidth and measurement signal frequency. It thus allows for separation in frequency from coupling parasitics, i.e., other frequencies present in the device under test, and makes the read-out quasi-noise-immune.

## 1. Introduction

Related research presents spectrometer systems based on optical Micro-Opto-Electro-Mechanical-System (MEMS, MOEMS) devices [1,2,3,4,5,6]. All of these developments aim at a miniaturized system, which is competitive regarding costs, easy to use, and still yields high measurement speed at comparable spectral resolutions. In [7], the authors suggested a novel mirror type and the so-called wishbone spectrometer structure. This approach is promising in terms of size and spectral resolution. However, it requires sophisticated process engineering and fabrication processes. In other approaches towards miniaturization of spectrometers, the Czerny–Turner structure has been proposed [8,9]. Although, it is well suited for miniaturization, this structure is very limited with respect to the achievable spectral resolution. In this work, we suggest to further miniaturize spectrometer devices by using rapid prototyping technologies to fabricate the housings, as well as the position sensor for the moving MEMS mirror. Instead of using optical position feedback, we suggest an inkjet-printed capacitive sensor and further also show the system’s noise immunity. Thus, the size of the device can be significantly reduced.

The MEMS mirrors employed in this work are already shock- and vibration-resistant [6] by design, which enables the operation of the resulting FTIR spectrometer in harsh environments. The micromirrors are operated at their mechanical resonance frequency to increase the drive efficiency. This provides another advantage in the spectrometer setup: the desired increased mechanical stroke or movement amplitude ($s_{m}=\pm 500\;\mu$m). Clearly, such miniaturized optical setups pose new research questions. They demand also for miniaturization of the mirror position measurement. In previous realizations, mainly optical position sensing for MOEMS was employed [5]. Such approaches, however, are large in comparison to what is possible using, e.g., a capacitive sensor. Consequently, we develop a small, flexible, and easy-to-fabricate sensor system (front-end and read-out) with high resolution and also high bandwidth capabilities. Since miniaturization of the spectrometer allows its usage on mobile platforms (e.g., mobile robots, drones), the developed system should be robust against changing environmental influences as well as immune against the high actuation voltages ($u_{exc}\approx 90\;V@f_{exc}=1\;\mathrm{kHz}$) at the electrostatic drive. Rapid prototyping technologies, such as 3D- and inkjet-printing, provide the necessary flexibility to produce optimized sensors that are immune to production inaccuracies (compare [10]). These technologies further enable cost-efficient devices in small series production, satisfying a major need for MEMS [11]. In addition, using metal 3D-printing, these designed packages are not only robust, but also functional in the sense that they inherently provide the shielding that is necessary for, e.g., a capacitive sensor.

Capacitive sensors are widely used in manifold applications [10,12,13,14]. Furthermore, capacitive sensing structures, electrodes, which basically are homogeneous layers of conductive material, are easier to fabricate (using, e.g., inkjet-printing) than coil structures. Additionally, also the resolutions, achievable with capacitive sensing, are well comparable to those achieved by inductive, optical, and other principles [15,16,17]. However, the bandwidth we can achieve with our system as a combination of high resolution hardware [18] and statistical signal processing [19] is better than those achieved by comparable systems considering the achieved spectral noise and sensor size [20].

**Figure 1:**
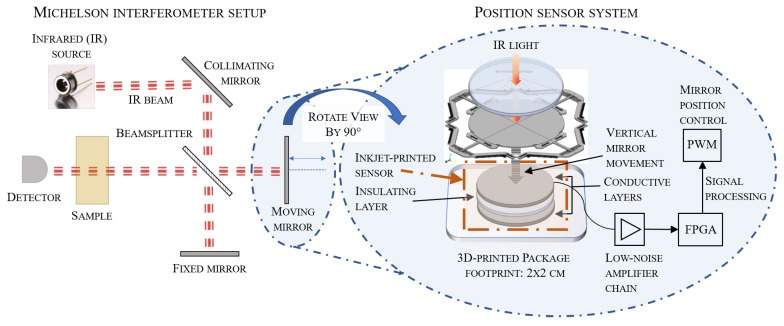
The basic construction principle of a Michelson interferometer is schematically illustrated. On the right, the suggested mirror position sensing system is shown. It is composed of a multilayer inkjet-printed capacitive sensor followed by a Low-Noise Amplifier (LNA) analog font-end and all digital signal processing on a Field Programmable Gate Array (FPGA).

A schematic of the developed architecture is illustrated in Figure 1. On the left, the Michelson interferometer setup employed is shown. On the right, a zoom of the moving MEMS mirror and the following hardware blocks are shown. The position measurement of this resonantly-moving mirror is fit into a maximum package outline of 2×2 cm.

## 2. Spectrometer System

Up to now, there has been a variety of literature on this topic [21,22], which presents elaborations of the spectrometer system similar to the one given in the following. A comprehensive analysis of spectrometry and related devices is given in [23].

Infrared spectrometry is a versatile measurement method mainly based on molecular vibrations. For large molecules and gases, also rotational components may be of interest and visible in the infrared spectrum. Additionally, FTIR spectrometry allows for analysis of samples in all phases of matter. Such devices rely on the interaction of light and matter, as can be analyzed using a so-called Michelson interferometer.

### 2.1. Michelson Interferometer

The Michelson two-beam interferometer principle is not the only useful setup to build an interferometer. Other designs have been developed and proven more appropriate for certain measurements. Nevertheless, the interferometer design as suggested by Michelson in the middle of the 19 th Century still best illustrates the working principle.

The beam of an infrared light source is split into two paths. These two paths are of different lengths and consequently cause a phase shift of the electromagnetic vectors of the two beams. At the subsequent recombination of these beams, interference occurs, the amplitude of which depends on the phase difference of the two beams. The light incident at the detector is then determined as a function of the path difference.

First, the collimating mirror collects and parallelizes the rays of the infrared light. Then, the beamsplitter, at the center of the apparatus, equally splits the incident light between the reference path (fixed mirror) and a path containing a mirror that moves perpendicular to the plane of incident light. The beams are reflected at the fixed and moving mirror surfaces and return to the beamsplitter. At the beamsplitter, both light beams interfere, yielding different intensities over all contained wavelengths depending on the different pathlength traveled. The interaction of the resulting beam with the sample then holds the spectral information, which is collected at the detector. One such reading is termed an interferogram. When the moving mirror is excited at constant velocity, the device is called a continuous-scan interferometer. This is in contrast to step-scan interferometers, which are equipped with stepwise moveable mirrors. Rapid-scan interferometers are a subclass of continuous-scan devices and rely on mirrors moving at velocities of $v_{m}\geq 0.1\;\mathrm{mm}/s$ [24].

### 2.2. Rapid-Scan Interferometry

**Figure 2:**
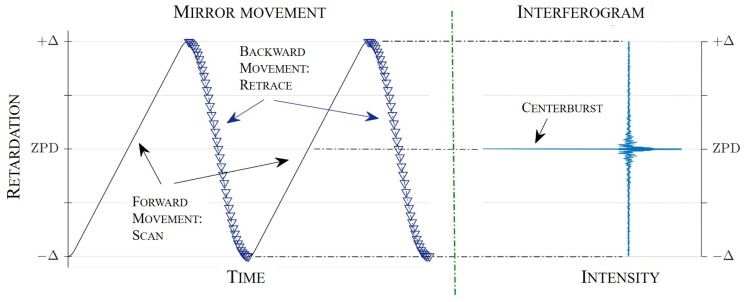
Illustration of the mirror movement for the acquisition of double-sided interferograms. The mirror is scanning only in the forward direction. The movement shape, which, in the illustration, deviates significantly form a proper sinusoid, will be properly sinusoidal for the considered micromirror application. In general, it will depend on the type of actuation and quality factor of the resonant device used.

Rapid-scan interferometers are devices with mirrors moving at constant velocities. To improve the SNR, it is common practice to record multiple interferograms and add them as part of an averaging process. The signal is added coherently, but the noise addition is non-coherent (under the assumption of randomly-distributed white noise). Thus, noise reduction can be achieved, and the SNR increases for *N* scans by a factor $\sqrt{N}$. In double-sided interferograms, the mirror movement starts at −Δ, crosses ZPDat the center of the path, producing the centerburst, and stops at +Δ. The process is illustrated in Figure 2: dependent on the mirror quality factor and actuation, the curve shape of the movement is more or less sinusoidal. In the case of the considered micromirror, the quality factor was comparably high. It can be determined as the ratio of the mirror center frequency and bandwidth, i.e., $Q_{mirror}=f_{c}/B\approx 1000$. Thus, the mirror movement will be properly sinusoidal, quasi independently of the type of actuation. Additionally, in the target application, the pathlength to be used for the generation of the interferogram will not extend to the top and bottom maximum values. Instead, the first and last ≈5% before/after the inflection points were not considered in order to avoid uncertainties introduced by non-homogenous mirror amplitudes.

### 2.3. MEMS Mirror

**Figure 3:**
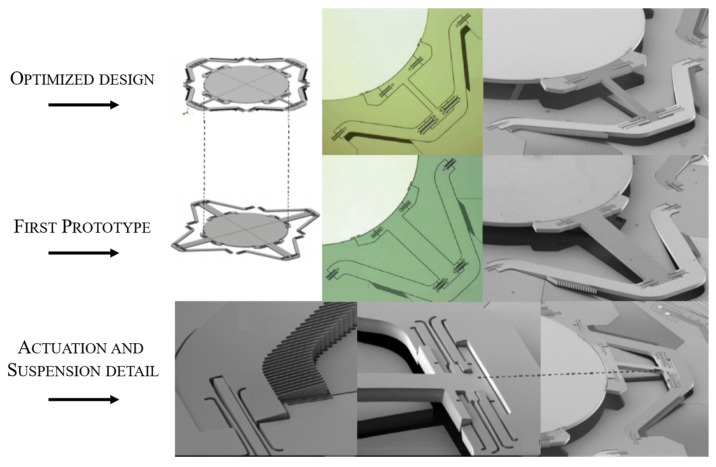
Graphical comparison of the preliminary and optimized mirror design, and details of the actuation and suspension. Adapted from [6] © SPIE 2014.

A graphical comparison of the preliminary and the optimized mirror design is given in Figure 3 with additional detailed photographs of the actuation electrodes and suspension. The considered MEMS-mirror device was manufactured in a CMOS-compatible Silicon On Insulator (SOI) process [25] with a layer thickness of 75 μm and an overall wafer thickness of 600 μm. The silicon layer was highly p-doped to produce a conductive surface, which was then coated with 50nm of aluminum, forming a reflective surface [26].

The target MEMS mirror was presented in [6]. It is a translatory MEMS with integrated electrostatic drives. Such devices are, in contrast to static mirrors, immune to shock and vibration, very fast, and highly compact. The target mirror is already an elaborate design, which should achieve a spectral resolution of $res_{W}=8\;{\mathrm{cm}}^{-1}$ over the mid-IR spectral range of λ=2.5−16 μm. Due to the resonance frequency of $f_{res}=500\;\mathrm{Hz}$, 500scans/s are possible together with high SNR>1000 [6]. Among others, the following specifications are given for the target mirror:

- resonance frequency $f_{res}=500\;\mathrm{Hz}$
- mirror diameter $d_{mirror}=5\;\mathrm{mm}$
- mirror movement relative to the rest position, i.e., the maximum stroke $s_{max}=\pm 500$ μm
- high reflectance at the wavelength of interest R≥95%
- low dynamic mirror deformation (warp) $w_{pp}<\lambda /10\;\mathrm{nm}$ (e.g., $\lambda =2500\;\mathrm{nm}\to w_{pp}<250\;\mathrm{nm}$)

To reduce parasitic (tilting) modes, the mirror was suspended on four pantograph-like structures, which were symmetrically arranged at the mirror rim. For the design with $d_{mirror}=5\;\mathrm{mm}$, a stroke of $s_{max}=\pm 500$ μm in a vacuum of 30Pa could be achieved. The target maximum warp of $w_{pp}=433\;\mathrm{nm}$ is larger than the target value. Reduction of the mirror diameter can reduce this maximum warp at the mirror rim. This is realized in a further optimized device with a diameter of $d_{mirror}=4.2\;\mathrm{mm}$. Also important is the actuation voltage of the system, which had to be kept below the pull-in voltage of the electrostatic actuation: the target mirror needs an actuation voltage of $v_{act}<110\;V$, which is below the pull-in voltage of $v_{\text{pull-in}}=118\;V$. This is necessary to guarantee stable operation of the electrostatic actuation. This actuation was realized by in-plane comb-electrodes, which were excited by a Pulse Width Modulation (PWM) signal (50% duty cycle) of twice the mirror’s oscillation frequency, and the voltage at the electrodes was typically between 40 and 90V.

## 3. Sensor System

**Figure 4:**
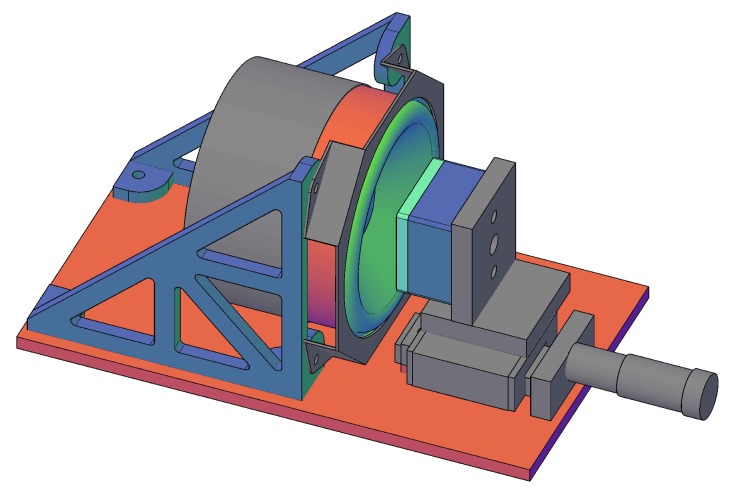
Demonstrator setup using the copper body on the linear stage (right-hand grey part) and loudspeaker (left-hand grey body with the membrane in green) mounted in parallel.

To evaluate the achievable noise and resolution of the printed sensor system experimentally, a demonstrator was set up (see Figure 4). The designed system was built to demonstrate the capabilities of the capacitive sensor. Thus, the system was tested in normal pressure using a loudspeaker. This loudspeaker was excited using an AC signal at the mechanical mirror resonance frequency. The membrane of the loudspeaker then moved in the same manner as the MEMS mirror. A piece of a bare wafer was attached to the surface of the membrane to simulate the mirror. In the following, first the design and fabrication are presented, followed by experimental results.

For the development of the demonstrator and also the final sensor housing, 3D-printing was employed. 3D-printing provides advantages such as fast development, easy re-design, and added functionality (e.g., as shielding for the capacitive sensor). The demonstrator consisted of a 3D-printed copper body, which held the inkjet-printed sensor structure. This copper body was then mounted to a linear stage, which was equipped with a micrometer screw to allow adjustment of the sensor position with an accuracy of $a_{srew}=50\;\mathrm{nm}$. The linear stage and the used loudspeaker were mounted on an aluminum plate. Both the sensor on the 3D-printed body and the loudspeaker with the wafer piece attached were mounted upright in a parallel position. To both, the conductive surface on the loudspeaker and the inkjet-printed sensor electrode, standard 50Ω cables were attached to connect the system to the designed hardware directly.

### 3.1. 3D-Printed Copper Body

**Figure 5:**
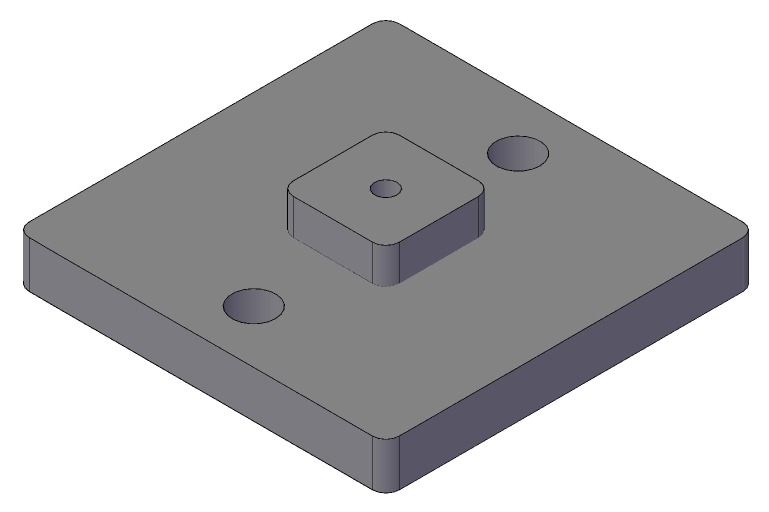
Illustration of the metal 3D-printed copper bodies, used as the substrate for the inkjet-printed capacitive sensor.

A 3D-printed copper body was used as a substrate for the inkjet-printed sensor. In a printing study [27], copper was identified as the best candidate to be combined with inkjet-printing. Two versions of the copper body were designed: the first version was designed to be used with the mirror brass package and was equipped with a ring, which enabled us to use an o-ring and proper mounting on the mirror package; the second version (Figure 5) did not have the mounting ring, but had an additional through-hole in the center to better connect the cable from the backside of the body. Additionally, two mounting holes were fabricated to fix the body. This second version of the copper body was designed for usage with the demonstrator system.

### 3.2. Inkjet-Printed Electrode

**Figure 6:**
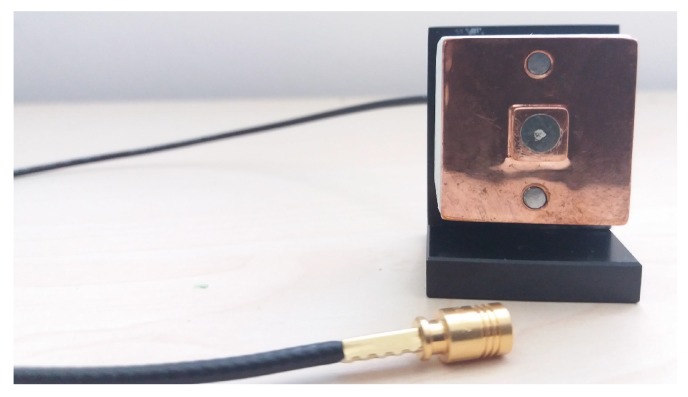
Illustration of the design of the copper housing and the printed electrode for the demonstrator setup; the electrical connection was realized through the central hole.

The inkjet-printed electrode in Figure 6 was fabricated as a multilayer structure. Two layers, an insulating and a conductive layer, had to be printed and cured subsequently. The 3D-printed copper body was sufficiently conductive and, thus at the same time, incorporated the shielding electrode necessary to reduce environmental influences on the measurement.

The inkjet-printing was done using the PiXDRO LP50 inkjet printer by Meyer Burger AG. This printer provides a dual-head assembly equipped with two identical inkjet print-heads (SM-128 Spectra S-class, Fujifilm Dimatix, Santa Clara, CA, USA) with a $d_{n}=50$ μm nozzle diameter and $s_{d}=50$ pL calibrated drop size. The print-heads were capable of a maximal resolution of $res_{max}=800\;\mathrm{dpi}$.

First, an insulating layer was printed to cover the copper at the center of the housing. The insulating, low-kdielectric ink was a mixture of acrylate-type monomers (Solsys EMD6200, SunChemical, Parsippany, NJ, USA). The dielectric ink was jetted at a head temperature of $t_{h}=50$ °C without substrate table heating. Then, conductive ink was printed to produce the electrode structure. The electrode was circular with a diameter of $d_{el}=5\;\mathrm{mm}$ in order to cover the whole mirror surface. In order to achieve a homogenous conductive surface, multiple layers of silver ink were printed and cured. At the electrode center, a through-hole was fabricated to connect a co-axial cable.

The conductive ink was a nanoparticle silver (Ag) ink (Sycris I50DM-119, PV Nanocell, Migdal HaEmek, Israel) with 50 wt% silver loading and an average particle size of $s_{p}=120\;\mathrm{nm}$ (d90). The silver ink was jetted at room temperature without heating of the substrate table. Drying the ink before photonic curing reduced the risk of outgassing. In this case, the silver ink was also part of a multilayer structure. It was printed onto insulating ink, which is not tolerant against elevated temperatures. Thus, substrate table heating was also avoided in this printing step. After each printing step, photonic curing, using specific UV-curing equipment, was done to finalize the printed structures.

Detailed analysis of the combination of 3D-printed substrates and inkjet-printing, as well as on the optimal choice of photonic curing parameters have been presented in [27].

The inner conductor of a co-axial cable was inserted into the through-hole of the electrode structure at the backside of the 3D-printed copper substrate, and its tip was mounted in plane with the inkjet-printed electrode structure. To assure a robust electrical connection between both, electrically-conductive silver adhesive was dispensed at the front, covering the interspace between the co-axial conductor and the electrode. The conductive adhesive can be seen as a light-grey circular structure at the electrode center in Figure 6.

An optimized design and how it affected the measurement outcome has been shown in [28].

### 3.3. Read-Out Hardware

The used read-out hardware was a laboratory prototype, which consisted of two major components: the analog front-end and the implementation of the digital signal processing. The hardware itself was based on a high-speed FPGA system with a customizable front-end. In the digital domain, the system was online configurable, i.e., without the necessity to rebuild the whole digital design each time certain parameters were changed.

#### 3.3.1. Analog Design

The underlying concept was a carrier frequency system where the capacitance of interest was series connected to a shunting resistor. Both components were driven by an AC voltage source, the voltage at the resistor was then measured using a low noise amplifier chain [20] and related to the capacitance change. We can thus significantly reduce the noise influence on the system and avoid, e.g., mitigation of noise into the electrical circuit [29].

To exert disturbing influences on such a system, the source of the disturbance needs higher energy compared to commonly-used architectures since the amount of transferred energy is generally higher at higher frequencies [30]. The impedance of the capacitance of interest was low in magnitude compared with parasitic resistances, which remained high independent of the frequency used. Therefore, the influence of the resistances was reduced and became neglectable at high enough frequencies. One disadvantage was the increased power consumption compared to other systems. Nevertheless, such a setup provided other benefits: parasitic capacitances can be positively included to from a bandpass filter for the suppression of unwanted bands.

**Figure 7:**
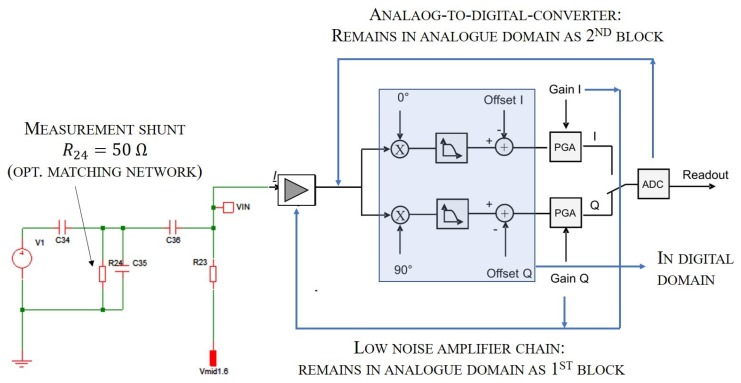
Read-out hardware architecture.

The high frequency signal was used as the excitation and will work as a carrier for the measurement signal. The capacitance of interest did an amplitude modulation of the carrier. This modulated signal was afterwards retrieved by demodulation. For the demodulation, different approaches are possible, out of which I/Q-demodulation was chosen. I/Q-demodulation provides high frequency selectivity, so that only disturbances in close vicinity of the carrier remain. Such a technique was further used for the hardware constructed. The specialty of the developed system (as illustrated in Figure 7) was now that we moved the majority of the components into the digital domain. Thus, we achieved significantly increased flexibility.

#### 3.3.2. Digital Signal Processing

The suggested FPGA-based hardware platform enables high sampling rates through the employment of high-speed ADCs and intelligent signal processing. FPGA design is also called hardware design, since usually, the programmed structure and behaviors are hard-wired, i.e., realized in hardware, and cannot be changed after building: to change the structure, rebuilding of the image is necessary, which may take up to a few hours for even moderately-sized designs. In the employed system, the blocks realized in the FPGA can be parametrized at runtime. These blocks are individually adaptable and thus enabled various system configurations without suffering from long compile times. Additionally, also the block connections are reconfigurable without the necessity to rebuild the image. It is thus possible to integrate and wire customized blocks in hardware (on the FPGA), as well as software (on the host computer).

This was further used to employ the necessary digital signal processing such as, e.g., demodulation, filtering, and offset corrections. Additionally, an Extended Kalman Filter (EKF) can be implemented. This was done to improve the hardware to yield a position resolution of up to $res_{pos}=20\;\mathrm{nm}$. The elaborated EKF held a sinusoidal system model and a measurement equation, which incorporated the inverse proportionality of the capacitance measurement [19]. To achieve higher frequency selectivity, a spread-spectrum approach may be used. For small series production, the necessary functionality can be ported to a basic FPGA device in a miniaturized hardware setup.

## 4. Experiments

**Figure 8:**
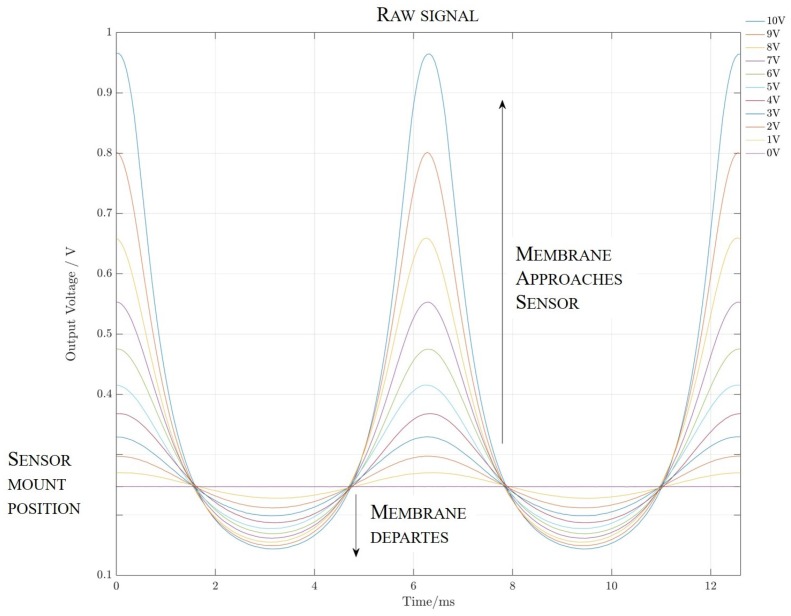
Raw measurement voltage signal.

First, the device response with varying measurement object amplitudes was evaluated. The printed sensor was placed at a distance of $d_{mem}=1\;\mathrm{mm}$ in front of the wafer piece on the membrane. Then, the membrane was excited at a frequency of $f_{mem}=160\;\mathrm{Hz}$. At this frequency, the maximum amplitude of its movement can be achieved. Then, the voltage at the membrane was switched in steps of $V_{step}=1\;V$ between $V_{min}=0\;V$ and $V_{max}=10\;V$. The resulting curves are shown in Figure 8. The highly non-linear behavior of the resulting signal is also obvious: the resulting curves when the membrane moved away from the sensor were compressed in comparison to the curves collected for the membrane approaching the mirror.

**Figure 9:**
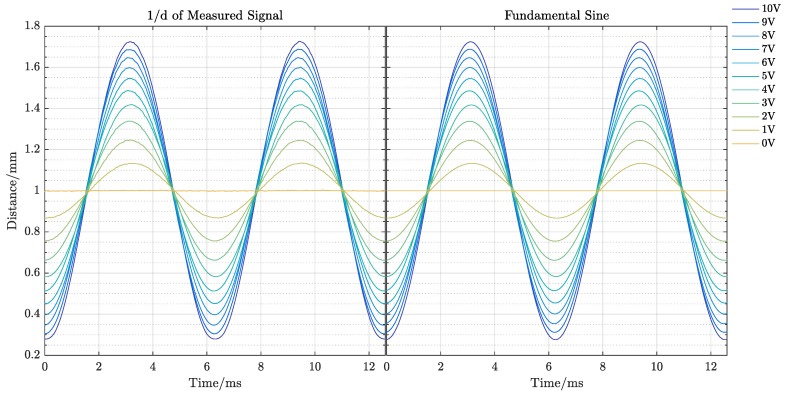
Comparison of the fundamental sine and sine determined from the reconstructed signal. The peaks as defined by the inverse of the curves where the membrane departs from the sensor are noisier than those measured in closer vicinity of the sensor.

In order to validate the assumed sinusoidal and inverse proportional characteristic in the EKF, the signal was first inverted. Then, a Fast Fourier Transform (FFT) was applied, and subsequently, all but the fundamental frequency was removed. The result was then subjected to an inverse FFT to determine the fundamental sine. This fundamental sine wave and the inverse of the measured signal are compared in Figure 9.

### 4.1. Hardware Resolution

**Figure 10:**
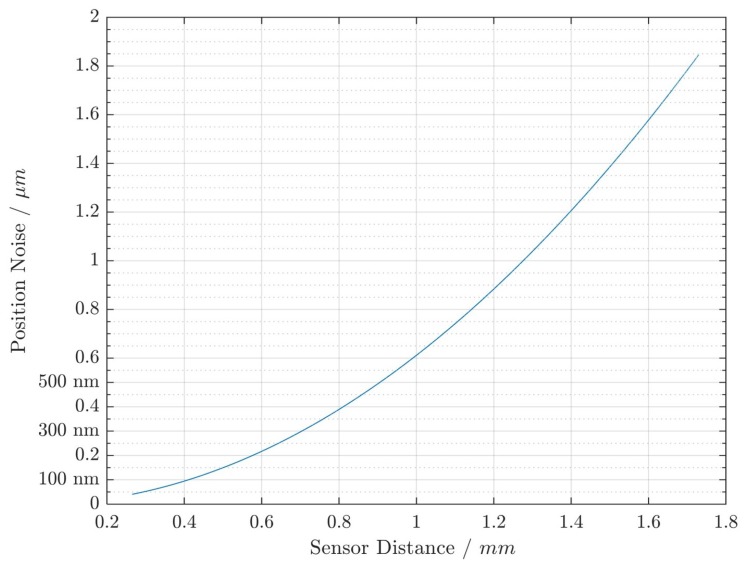
Illustration of achieved position noise dependent on the sensor distance using a bandwidth of $B_{meas}=10\;\mathrm{kHz}$.

In order to determine the resolution of the sensor dependent on the distance, it is necessary to determine its sensitivity as a function of the distance. The sensitivity of the sensor as dependent on the distance is the respective derivative of the raw signal, as shown in Figure 8. This was done numerically for the given measurement data. The resolution was then determined by multiplying the derivatives by the sensor noise floor (the capacitance equivalent noise was $C_{n}=151\mathrm{zF}/\sqrt{\mathrm{Hz}}$ [20]). The resulting sensor resolution capability over distance is given in Figure 10.

At sensor distances below $d_{mem}=0.4\;\mathrm{mm}$, we see that position resolutions better than $res_{pos}=100\;\mathrm{nm}$ were possible. The more the sensor’s distance to the measurement object increases, the less the position resolution becomes. In order to counteract this nonlinear phenomenon, the usage of an EKF as mentioned in Section 3.3.2 is suggested.

### 4.2. Disturbance Rejection

**Figure 11:**
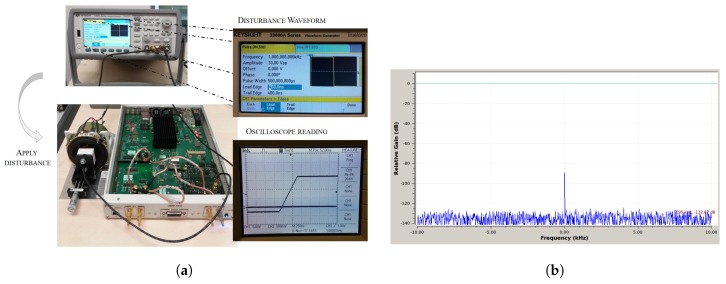
Disturbance evaluation setup and resulting FFT. (**a**) Measurement setup used for the disturbance evaluation. (**b**) FFT of the received signal with disturbance signal applied at the sensor counter electrode.

In the considered application for position sensing of the MEMS mirror, a known source of disturbance is the large mirror excitation signal. The developed carrier frequency sensing concept was designed to be unaffected by the mirror excitation. In order to demonstrate this, a PWM, i.e., rectangular, signal of $f_{dist}=1\;\mathrm{kHz}$, and an amplitude of $u_{dist_{pp}}=20\;V$ peak-peak, was connected to the sensor’s counter electrode (Figure 11a). Then, the FFT of the received signal was observed (see Figure 11b).

In the resulting FFT, there was no other signal peak than the carrier: obviously, disturbing influences, such as the mirror excitation signal, were eliminated through the developed architecture. Furthermore, in the final system configuration, the excitation signal was not directly at the sensor’s counter electrode, but at the mirror rim (where the driving electrodes are; compare the comb structure as shown in Section 2.3).

However, restrictions have to be made for the edge steepness of the PWM, where signals with higher change rates than $r_{c}=25$ V/μs caused saturation of the analog amplifier chain.

## 5. Conclusions

A printed capacitive sensor system, applied to an MEMS mirror in an FTIR spectrometer, is designed, built, and experimentally evaluated with respect to noise and disturbance rejection. The considered hardware holds a custom analog circuitry attached to an FPGA to provide flexible signal processing. This hardware is flexibly applicable also to other sensing principles. A discussion of the interferometer principle and rapid-scan interferometry is given. The sensing system is discussed giving details on the target MEMS mirror, as well as an illustration of the 3D-printed copper housing and details on the inkjet-printing process to fabricate the capacitive sensor electrodes.

The presented experiments illustrate the high achievable position resolution of $res_{pos}=20\;\mathrm{nm}$ at high bandwidths (here, B=10kHz) and disturbance immunity of the built system. Consequently, we show that the implementation of all-printed capacitive sensors for nanometer position resolutions and high bandwidth is feasible also for miniaturized setups. The addition of smart statistical signal processing enables further improvements. We further show how a robust analog front-end for capacitive sensors can be designed and outline the high achievable noise immunity by using the proposed setup. Additionally, instead of changing the spectrometer architecture, we show how the employment of rapid prototyping technologies can be used for miniaturization. This also clearly distinguishes this work from the current state-of-the-art. Exploiting the flexibility of the inkjet-printing used for optimized electrode design as part of further work can additionally reduce the measurement error.
